# Emissions Reductions at Coal Power Plants Continue to Offer Routes to Meet New US PM_2.5_ Standards

**DOI:** 10.1021/acs.est.5c13420

**Published:** 2026-05-05

**Authors:** Munshi Md Rasel, Daniel S. Cohan, Daniel Tong, Lucas R. F. Henneman

**Affiliations:** † Department of Civil, Environmental, and Infrastructure Engineering, 3298 George Mason University, Fairfax, Virginia 22030, United States; ‡ Department of Civil and Environmental Engineering, 3990 Rice University, Houston, Texas 77005, United States; § Department of Atmospheric, Oceanic and Earth Sciences, 3298 George Mason University, Fairfax, Virginia 22030, United States

**Keywords:** coal power plants, PM_2.5_, National Ambient Air Quality Standards, air pollution health impacts

## Abstract

The 2024 revision of the National Ambient Air Quality Standard for particulate matter less than 2.5 μm diameter (PM_2.5_) to 9 μg/m^3^ by the US Environmental Protection Agency (EPA) has motivated an assessment of whether reducing Electric Generating Units (EGU) emissions is a potential strategy for bringing nonattainment counties into compliance. We assessed coal power plants’ contributions to PM_2.5_ using a chemical transport model. We identified the contribution of specific coal EGUs to PM_2.5_ concentrations using a reduced complexity air quality model and demonstrated health benefits of emissions reductions at facilities that would need to shutter to attain the new standard. In 2023, 9 nonattainment counties could have met the standard by eliminating SO_2_ emissions at 94 facilities (9% of US EGU capacity). Retiring these EGUs would avoid stack emissions of 500,000 tons of SO_2_, 304,000 tons of NO_*x*_, and 485 million tons of CO_2_, along with approximately 1,170 premature deaths per year (95% confidence interval: 1,060–1,280) among elderly people. Reducing coal power plant emissions continues to provide an avenue to meet U.S. air quality standards and improve public health.

## Graphical abstract

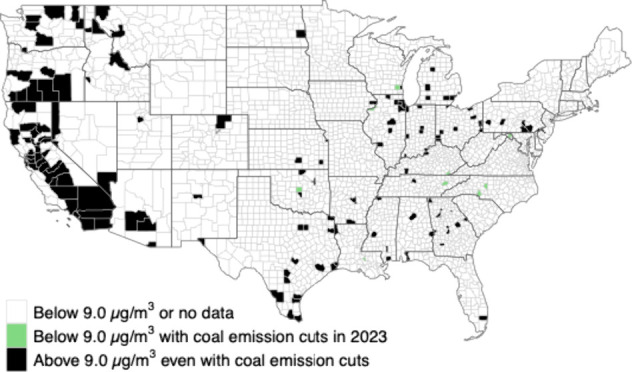

## Introduction

In 2024, the United States Environmental Protection Agency (EPA) promulgated a new health-based National Ambient Air Quality Standards (NAAQS) for PM_2.5_, set at 9 μg/m^3^.^1^ This standard sparked legal challenges from several states, which argued that the EPA has disregarded the economic implications of setting these stringent PM_2.5_ standards, even though the Clean Air Act (CAA) states that NAAQS should be health-based.^2^ Additionally, the EPA issued new regulations for greenhouse gas emissions from coal-fired Electric Generating Units (EGUs).^3^ Since these actions, the current administration has announced plans to “release guidance to increase flexibility on NAAQS implementations”^4^ and proposed a replacement rule that would repeal all greenhouse gas emissions standards for fossil-fuel EGUs.^5^ The administration has taken several actions to delay coal power plant retirements, including through emergency declarations. The controversy over EGU regulations motivates an investigation into the potential for emissions reductions at coal power plants to improve human health and help PM_2.5_ nonattainment areas achieve the NAAQS.

Historically, coal power plants were significant sources of ambient PM_2.5_ in the eastern United States, with smaller contributions to PM_2.5_ in the West.^6–8^ Regulations such as the Acid Rain Program, the Clean Air Interstate Rule (CAIR), and the Cross-State Air Pollution Rule (CSAPR) compelled coal power plants to use low-sulfur fuel, install emissions control devices, and/or shut down entirely.^9–11^ Reduced economic activity during the 2008 recession and the economics of natural gas resulted in further reductions in coal power plant emissions and associated human exposures and deaths.^12^ By 2020, coal power plant SO_2_ emissions contributed less than 1 μg/m^3^ to annual average PM_2.5_ in most of the United States, and EGU SO_2_ emissions were linked to 1,600 deaths in the Medicare population in 2020, down from an annual average of 43,000 per year from 1999 to 2007.^7^ Due to the poor profitability of existing coal power plants, further reductions in emissions are likely to come from retirements instead of costly emissions control technology retrofits.^3,13^

EPA’s regulatory impact analysis for the 2024 PM_2.5_ NAAQS investigated emissions reductions needed to meet the standards in 2032.^14^ In its analysis, EPA predicted PM_2.5_ concentrations in 2032 that assumed that coal power plant retirements would remain consistent with documented retirement dates and regulations promulgated as of 2022. However, the evolving regulatory environment surrounding coal EGUs has increased uncertainty surrounding EPA’s original projections. States containing nonattainment areas will be compelled to develop state implementation plans (SIPs), which may or may not dictate shuttering or installing controls on power plants.

We present an investigation into power plants’ influence on PM_2.5_ in each contiguous U.S. county in which the PM_2.5_ design value reported by the EPA exceeded 9 μg m^–3^ in 2023. We identified the coal EGUs that would need to retire to bring these areas into attainment. Finally, we quantified the potential avoided SO_2_, NO_*x*_, and CO_2_ emissions from these EGUs and the premature mortalities attributable to their SO_2_ emissions.

## Methods and Materials

We assessed the contributions of SO_2_ emissions from all EGUs to total PM_2.5_ using the Community Multiscale Air Quality (CMAQ) model. We focus on SO_2_, a precursor of secondary PM_2.5_, because it is well established that SO_2_ is the dominant pollutant contributing to PM_2.5_ health damages from United States power plants (relative to NO_*x*_ or primary PM_2.5_).^15–18^ We then identified the most impactful coal EGUs with a dispersion-based reduced complexity model, HYSPLIT Average Dispersion Model (HyADS).

### Chemical Transport Model

Using CMAQv5.4, a chemical transport model developed by EPA^19^ with the first order direct decoupled method (DDM),^20–22^ we quantified gridded air pollution concentrations and their sensitivities to EGU SO_2_ emissions over a 12 × 12 km^2^ domain covering the contiguous U.S. in 2020 (Figure S1). We quantified PM_2.5_ sensitivities to all EGU SO_2_ emissions, of which 95% came from coal EGUs.

CMAQ emission inputs were processed using the Sparse Matrix Operator Kernel Emissions (SMOKE) model. Emissions were based on the 2017 National Emission Inventory data adjusted for the effects of the COVID-19 interventions in 2020. Year 2020 EGU emissions reported to EPA were used directly. Detailed information on the emission data generation process can be found in Campbell et al. (2021).^23^ Meteorological inputs were generated with the Weather Research & Forecasting (WRF) model version 4.4,^24^ adopting default parameters from the WRF user guide.^25,26^

We used CMAQ-DDM to quantify the first-order impact of EGU SO_2_ emissions on ambient PM_2.5_ concentrations. DDM calculates sensitivity coefficients of all species concentrations using the Advection Dispersion Reaction (ADR) equation with respect to specified model inputs or parameters.^27^ A perturbed model parameter (normalized model input parameter) *p*_*j*_ (*x,t*) can be calculated from their unperturbed parameters (non-normalized model input parameter) *P*_*j*_ using a scaling factor, ∈_j_:

1 $${p_{j}=\in }_{j}P_{j}$$

The local sensitivity, also known as a seminormalized sensitivity coefficient, of concentration response to *p*_*j*_ (here for example, emissions) can be measured by scaling the non-normalized local sensitivity coefficient of the unperturbed value *p*_*j*_ of the parameter:

$$S_{\mathrm{ij}}\left(t\right)=P_{j}\frac{\partial C_{i}\left(t\right)}{\partial p_{j}}=P_{j}\frac{\partial C_{i}\left(t\right)}{\partial \left(\in_{j}P_{j}\right)}=\frac{\partial C_{i}\left(t\right)}{\partial \in_{j}}$$ 2

In eq 2, *S*_*ij*_(*t*) is the seminormalized sensitivity coefficient representing change in concentration of species *i* at time *t* with respect to parameter *p*_*j*_. *S*_*ij*_(*t*) are in the same units as *C* and provide first-order (linear) estimates of the responsiveness of concentrations infinitesimal changes in parameters, scaled to a 100% reduction in the parameter (here, EGU SO_2_ emissions).

### Quantifying Facility-Specific PM_2.5_ Contributions to Each County and Changes over Time

To identify the EGUs that contributed to PM_2.5_ in each county, we used the HYSPLIT with Average Dispersion (HyADS) model.^28^ HyADS employs the HYSPLIT transport and dispersion model to track the movement of air parcels.^29^ HYSPLIT tracks 100 air parcels from each facility four times daily for 7 days. These air parcels are then aggregated into a 36-km grid and weighted by each EGU’s monthly SO_2_ emissions. The emissions-weighted concentrations of these air parcels are converted to PM_2.5_ concentrations using a month-specific statistical linear regression model derived using output from a full-complexity chemical transport model.^28^ Stack height data for HyADS were sourced from the EPA’s 2017 National Emission Inventory.^30^ EGU emissions data were obtained from the EPA’s Air Markets Program Data.^31^

HyADS replicates spatial patterns and magnitudes of PM_2.5_ attributable to SO_2_ emissions from individual coal EGUs, as evidenced by comparisons with more complex air quality models.^32^ Evaluations, however, suggest HyADS may underestimate total PM_2.5_ from coal SO_2_ emissions in recent years, likely because it assumes year 2005 atmospheric photochemical state.^7^ SO_2_ and NO_*x*_ emissions were higher in 2005 compared to 2020, but emissions of NH_3_, a third precursor for secondary inorganic PM_2.5_, remain abundant.^34^ Therefore, observed changes in PM_2.5_ sensitivity to EGU SO_2_ emissions due to precursor emissions are not considered by HyADS. Therefore, we used HyADS only to estimate the fractional contribution of each EGU to the total contribution from all coal EGUs and their changes over time. To estimate each EGU’s contribution to PM_2.5_, we multiplied its fractional contribution in each county from HyADS by the total CMAQ-DDM PM_2.5_ sensitivity to EGU SO_2_.

HyADS facility-specific PM_2.5_ contributions and CMAQ-DDM output were computed for year 2020. To estimate each EGU’s contribution to PM_2.5_ in years 2021–2023 (aligning with the most recent release of EPA’s eGRID data, which is described below), we scaled each facility’s PM_2.5_ contributions by the ratio of that facility’s year-specific SO_2_ emissions to the year 2020 emissions. We scaled CMAQ-DDM EGU PM_2.5_ in each county by the ratio of year-specific HyADS to the comparable HyADS value in 2020. This approach follows the linear scaling in HyADS, but it does assume constant atmospheric transport across years. Gridded HyADS and CMAQ-DDM output are assigned to counties by area weighting—i.e., grid cell concentrations are weighted to counties by the portion of the county covered by each grid cell.

Design values are calculated by EPA as the 3-year average PM_2.5_ rounded to the nearest 0.1 μg m^–3^. Accordingly, we calculated EGU contributions to each county’s design values as the 3-year average PM_2.5_ from EGUs. Using these EGU PM_2.5_ contributions and actual EPA design values in year 2023, we identified which counties with design values greater than 9.0 μg m^–3^ would have achieved the standard without coal EGU SO_2_ emissions (i.e., their design values were less than 9.0 μg m^–3^ after subtracting coal EGU PM_2.5_ contributions). In a supplemental analysis, we repeated the analysis for coal EGU impacts and design values in other years (design value years 2020–2024).

### Identifying the Most Impactful EGUs on Each County

To estimate the EGUs that need to retire to attain the standard in each county, we rank-ordered EGUs by their annual average contribution to PM_2.5_ in that county in 2023. Starting with the most impactful EGU and proceeding down the rankings, we identified the EGUs that would need to retire before the county just met the 9 μg m^–3^ standard. This approach assumes the most impactful EGUs would be targeted for emissions reductions first. Consistent with existing evidence,^3,13^ we assume that future SO_2_ emission reductions will occur through facility closures instead of end-of-pipe controls such as scrubbers. We report retirement plans based on information available in year 2023 eGRID.^35^

### Quantifying Cobenefits of Meeting the PM_2.5_ Standard

Benefits and costs of shuttering power plants extend beyond the nonattainment counties themselves. For the EGUs that would need to retire for each nonattainment county to achieve attainment, we calculated 1) their total nameplate capacity to understand the amount of electricity generation that would need to be replaced;^35^ 2) annual CO_2_, NO_*x*_, and SO_2_ emissions in 2023; and 3) avoided deaths in the Medicare population from reduced PM_2.5_ exposure specifically associated with coal EGU SO_2_ emissions. The cobenefits are relative to the year 2023 baseline.

To estimate potential Medicare deaths avoided, we relied on a modeling system that integrated HyADS exposures with Medicare health data.^7^ Medicare deaths attributable to each coal EGU are quantified using concentration response function between HyADS exposures and deaths in the Medicare population. This approach implicitly accounts for uncertainties in HyADS-modeled PM_2.5_ contributions from coal EGUs, as the concentration response function was derived using the HyADS exposure data for the population of interest. For each unit that would need to retire to enable a county to meet the NAAQS, we calculated potential deaths avoided as the number of deaths associated with that unit in year 2020 (the most recent year available) scaled by the ratio of 2023 SO_2_ emissions to 2020 SO_2_ emissions. While this scaling does introduce uncertainty, Henneman et al. (2023) reported nearly linear relationship between emissions and associated mortality within time spans of a few years even when considering meteorological variability. Henneman et al. (2023) found that the number of Medicare deaths associated with coal EGUs may be underestimated in recent years due to the potential underestimation of PM_2.5_ from EGU SO_2_ emissions, so values reported here should be considered conservative. Additionally, the Medicare population only represents U.S. citizens age 65 and older, so mortalities in other populations are not accounted for in this analysis.

## Results and Discussion

In this section, first we provide an overview of CMAQ-modeled PM_2.5_ concentrations and sensitivities to SO_2_ emissions from all EGUs. Second, we identify the counties that can be brought into attainment by controlling coal EGU emissions. Finally, we present the cobenefits that can be achieved by controlling coal EGUs needed to bring counties into attainment.

### PM_2.5_ Sensitivity to Coal EGU SO_2_ Emissions

We evaluated CMAQ-modeled daily PM_2.5_ concentrations against EPA AQS ground observation data and find the performance meets criteria standards from Emery et al. (2017)^36^ for Normalized Mean Bias (NMB), Normalized Mean Gross Error (NMGE), and Root Mean Square Error (RMSE) in most region-season pairs (Supporting Information, including Figures S1–S4). CMAQ-modeled annual average PM_2.5_ concentrations are highest in the eastern and midwestern U.S., particularly in the Ohio River Valley, the Northeast, and parts of the Southeast (Figure 1). These areas experience elevated PM_2.5_ concentrations primarily due to a combination of coal-fired EGU emissions, industrial activities, and transportation, in alignment with EPA’s regional PM_2.5_ trend assessment.^37–39^ Outside of local hotspots like California’s South Coast Air Basin and San Joaquin Valley, the western United States generally shows lower PM_2.5_ concentrations compared to the East.

**Figure 1:**
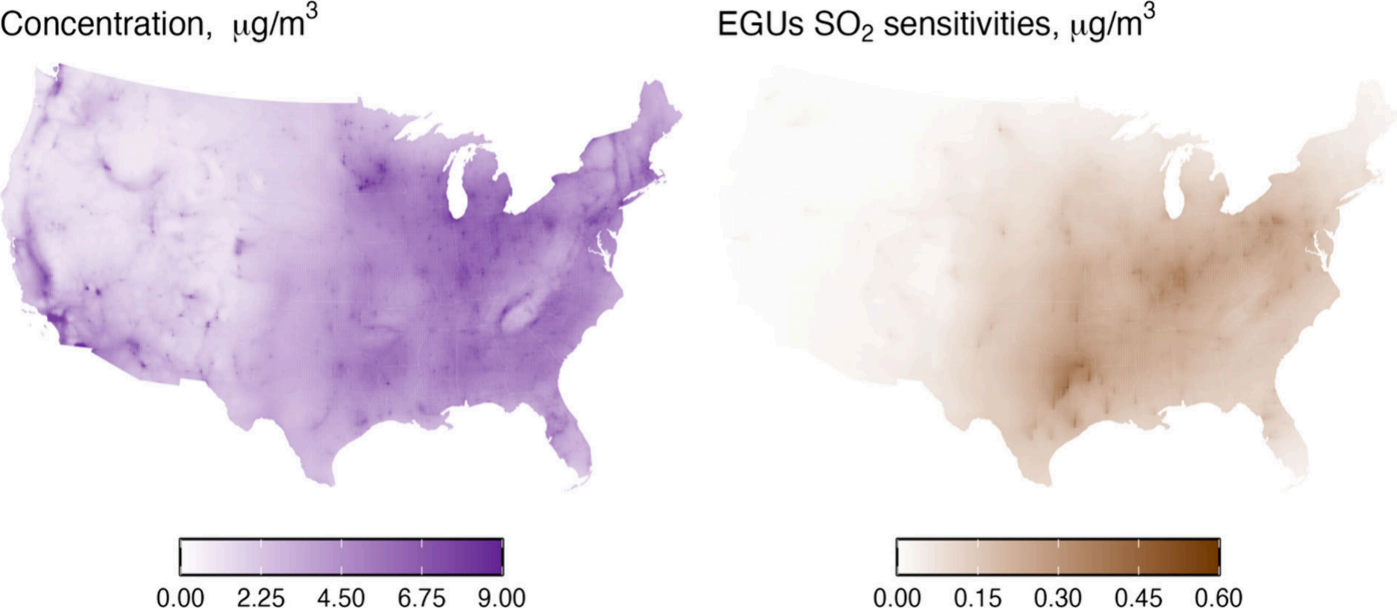
Annual average PM_2.5_ concentration (left) [range: 0.9 to 16 μg/m^3^] and average PM_2.5_ sensitivity [range: 0.0 to 0.5 μg/m^3^] to EGU SO_2_ emissions (right) in 2020.

PM_2.5_ sensitivities to EGU SO_2_ emissions are greater in the East than the West, with the largest magnitudes over eastern Texas and along the Ohio River Valley (Figure 1), aligning with locations of remaining US coal EGUs. From these maps and locations of coal EGUs (Figure S1), it is anticipated that reductions in SO_2_ emissions would have the largest impacts on PM_2.5_ concentrations in these areas. Conversely, the western U.S. shows lower sensitivity to EGU SO_2_ emissions, indicating that PM_2.5_ in this region is from sources other than EGUs. Annual average PM_2.5_ sensitivities to coal EGU SO_2_ emissions vary by greater than an order of magnitude across regions—the median is 0.09 μg/m^3^ and the interquartile range (IQR) spans from 0.02 μg/m^3^ to 0.2 μg/m^3^. The maximum sensitivity observed is 0.5 μg/m^3^, which occurs in the industrial Midwest.

### Coal EGU Contributions To Be in Attainment

Out of 3,109 counties in the contiguous US, EPA reported that 157 counties had design values above the 9 μg/m^3^ PM_2.5_ standard in 2023.^14^ We identified nine counties in 2023 whose design values could be brought below the standard by eliminating SO_2_ emissions from operating coal EGUs alone (Figure 2).

**Figure 2:**
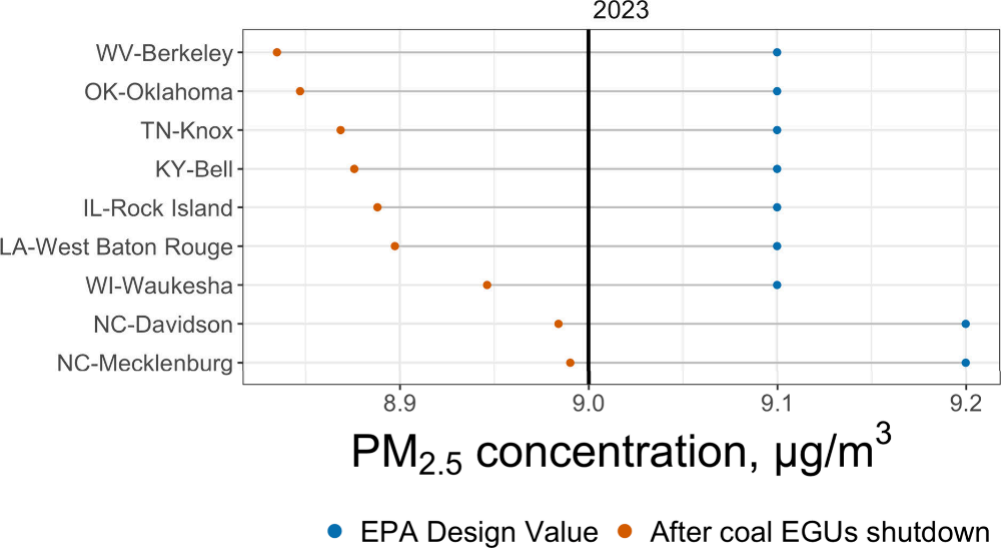
Year 2023 observed and expected design values with a 100% reduction in EGU SO_2_ emissions as calculated from the year 2020 CMAQ-DDM sensitivities adjusted to years 2021–2023 by the ratio of facility-specific SO_2_ emissions. Counties shown are restricted to those that could attain the 9.0 μg/m^3^ standard through EGU SO_2_ emissions reductions alone.

Reducing EGU SO_2_ emissions would primarily benefit counties in the central and eastern US because of the location of the remaining large coal power plants and prevailing wind patterns (Figure 3). Counties located in the western US would benefit very little from reducing coal emissions. Additional measures beyond controlling EGU SO_2_ emissions are necessary for counties beyond those we have identified to reduce their design values below 9 μg/m^3^. The list of counties that could achieve the standard through EGU emission cuts alone varies between years since it is dependent on year-specific PM_2.5_ from coal EGUs and the amount that each county exceeds the 9.0 μg/m^3^ standard (Figure S5).

**Figure 3:**
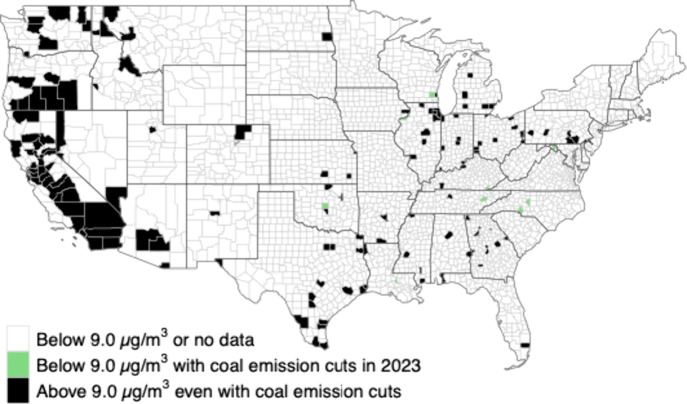
U.S. counties that either are in attainment or do not have data, can achieve attainment through eliminating coal EGU SO_2_ emissions alone, or cannot achieve attainment through eliminating coal EGU SO_2_ emissions alone.

CMAQ-DDM is uncertain, and employing first-order sensitivities tends to estimate slightly larger PM_2.5_ sensitivities than indicated by a linear scaling of the responsiveness to an infinitesimal change (first-order DDM sensitivities used here do not consider nonlinearities in gas-aerosol partitioning of NH_3_ and NH_4_).^40–42^ The scaling to years other than 2020 potentially introduces additional uncertainty. In a sensitivity analysis, we applied a global uncertainty range of 50% to 150% to our PM_2.5_ sensitivities and identified the counties that could meet attainment through **SO**_**2**_ emissions reductions from all EGUs (Figure S6). This sensitivity analysis revealed some differences in which counties may be able to meet the standards by eliminating SO_2_ emissions from coal EGUs. If CMAQ-DDM overestimates PM_2.5_ sensitivities from EGUs by 50% (i.e., actual sensitivities are half as large as modeled), 6 counties could achieve attainment by controlling EGU SO_2_ emissions. In contrast, if actual sensitivities are 1.5x as large (i.e., CMAQ-DDM underestimates actual sensitivities by 50%), we find that 20 counties could be brought into attainment by controlling all EGUs’ SO_2_ emissions in 2023.

### Cobenefits of Eliminating EGU SO_2_ Emissions for NAAQS Attainment

Retiring coal EGUs to bring nonattainment counties into attainment would result in substantial emission reductions and other benefits and costs (Figure 4). Bringing all nine counties into attainment by closing coal EGUs would require eliminating emissions from 94 facilities. Emissions from these facilities represent 77% (500,000 tons), 46% (304,000 tons), and 31% (486 million tons) of total US EGU SO_2_, NO_*x*_, and CO_2_ emissions, respectively.

**Figure 4:**
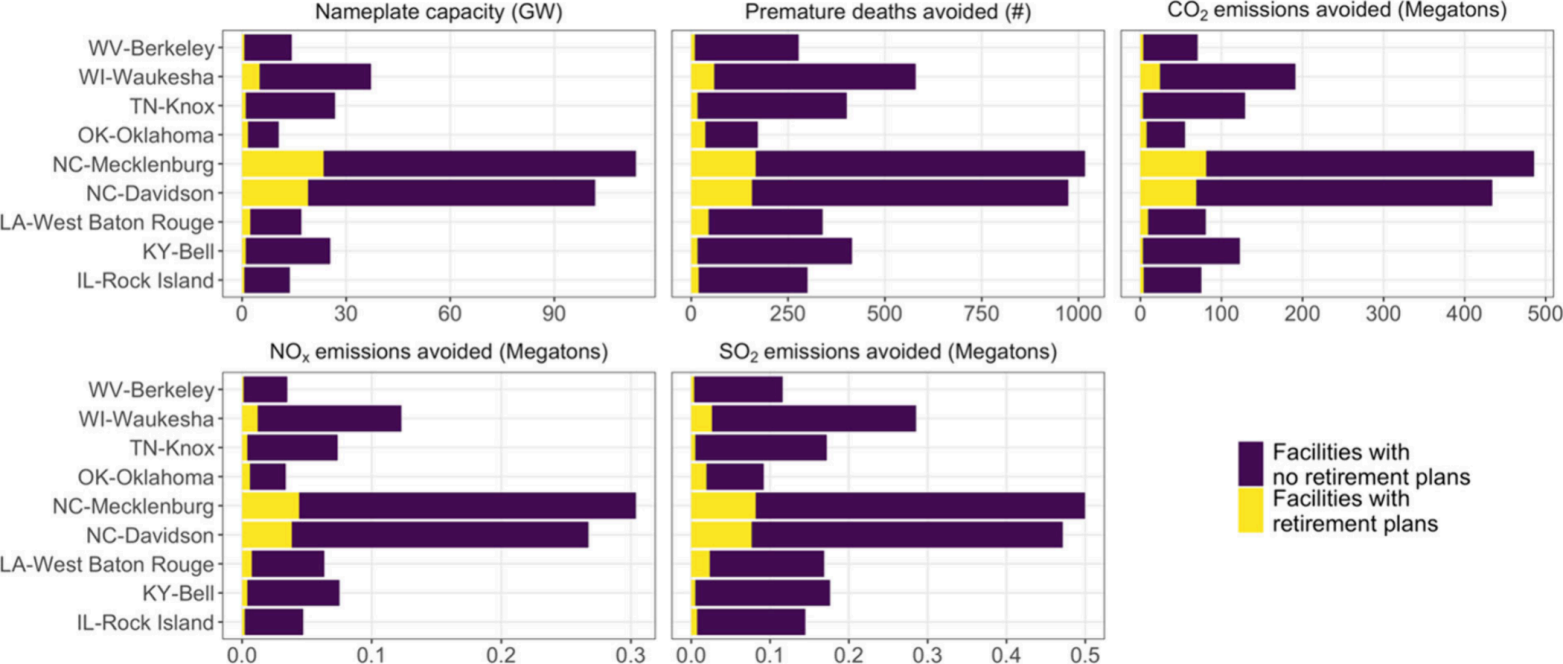
Summary statistics corresponding to the smallest number of U.S. coal EGUs that would need to shutter to attain the NAAQS in each U.S. counties. Information includes total nameplate capacity; number of annual Medicare deaths (age 65 and older) that will be avoided across the US; and avoided CO_2_, NO_*x*_, and SO_2_ emissions from the coal EGUs that would retire. Facility retirement plans refer to documented plans by 2032 in eGRID (2023).^35^ EGUs may be located outside their corresponding county.

Facilities summarized in Figure 4 represent the smallest number of facilities that would need to shutter to attain the PM_2.5_ NAAQS in each county, and the facilities are not necessarily located in that county. The number of coal EGUs that would need to retire to meet attainment varies between counties. For example, bringing Knox County, TN into attainment by shuttering coal EGUs would require closing 13 facilities (Figure 4). Among these facilities, one has documented plans to retire all of its coal capacity by 2032.^35^ In 2023, these 13 facilities emitted 172 thousand tons of SO_2_, 73 thousand tons of NO_*x*_, and 129 million tons of CO_2_. Bringing two North Carolina counties into attainment would require closing far more facilities than the other counties (94 for Mecklenburg and 79 for Davidson) because attaining the standard would require eliminating nearly all of the coal EGU PM_2.5_ contributions to these counties (Figure 2).

The public health benefits in terms of avoided annual premature death for people 65 years and older from the 94 coal EGUs’ SO_2_ emissions totals 1,170 premature deaths per year (95% confidence interval: 1,060–1,280) deaths per year. Potential health benefits from attaining the standard in each county depend on emissions and proximity of the facilities to population centers (Figure 4). There is large overlap between facilities identified across counties.

While the health impact analysis presented here focuses on the Medicare population (age 65 years and older), substantial evidence indicates that reductions in PM_2.5_ exposure also confer important health benefits for younger populations. Epidemiological studies have linked long-term PM_2.5_ exposure to increased all-cause mortality among adults under age 65, as well as elevated risks of cardiovascular disease, asthma incidence and exacerbation, adverse birth outcomes, and impaired lung development in children.^43–46^ Similarly, researchers have linked installations of emissions control devices at coal plants and coal power plant closures specifically to asthma outcomes in a population of all ages^47^ and birth outcomes.^48^ Consequently, the total public health benefits of coal EGU retirements are likely larger than the estimates reported here, which should be interpreted as conservative lower-bound estimates of overall health impacts.

Closing coal EGUs would reduce the available electricity generated from these plants. Retiring the coal-fired EGUs identified here would require consideration of grid reliability and replacement capacity, particularly in regions where coal plants provide dispatchable power. Figure 4 summarizes capacity associated with facilities contributing to PM_2.5_ nonattainment in specific counties, but coal retirements would be managed at the grid balancing regional level rather than solely at the county or state level. In total, the facilities comprising the 94 EGUs have a nameplate capacity of 113,500 MW (9% of total capacity in 2023 [1,260,000 MW]; Figure 4 & Table S1). Capacity is calculated from eGRID on facilities with larger than 0 capacity factor. Assuming constant of growing electricity demand, this is generation capacity that would need to be replaced—in the three largest grid balancing regions, for example, facilities identified by this analysis represent 8% of ERCOT (Texas) capacity, 13% of MISO (covering much of the midwestern US) capacity, and 13% of PJM (covering parts of the Mid-Atlantic) capacity. Capacity factors (i.e., the fraction of actual output vs the capacity) of these facilities averaged 43%, slightly higher than the 40% for all facilities.

In practice, replacing retired coal capacity would likely involve a portfolio of resources, including natural gas combined-cycle generation, energy storage, transmission expansion, demand-side management, and renewable generation.^49^ Similar coal retirements over the past decade have been accommodated without major reliability disruptions through coordinated planning by grid operators, suggesting that the retirements examined here are technically feasible, though each would require deliberate capacity planning and investment.

Climate benefits suggested by potential CO_2_ emission reductions should be interpreted relative to emissions of potential replacement technologies. While a full life-cycle assessment of replacement generation technologies is beyond the scope of this study, the literature provides estimates of life-cycle emissions from major electricity generation sources.^50^ Coal-fired electricity generation (820 g CO_2_eq/kWh [IQR: 740–910]) is associated with approximately double the life-cycle CO_2_ equivalent emissions compared to combined-cycle natural gas (490 [IQR: 410–650] g CO_2_ eq kWh^–1^), and over an order of magnitude more emissions than wind (11 [IQR: 7–22] g CO_2_ eq kWh^–1^) and utility-scale solar (48 [IQR: 20–80] g CO_2_ eq kWh^–1^).^50–52^ Multiplying these emission factors by generation associated with the coal facilities identified in this analysis (444 million MWh) suggests that replacing coal generation with currently deployed alternatives would yield net reductions of about 150 million metric tons of CO_2_ per year for natural gas replacement, and between 300 and 400 million metric tons of CO_2_ per year for wind or solar replacement. Construction of new capacity (including renewables) could lead to air pollution at levels relevant to public health,^53^ and new natural gas capacity would lead to air pollution emissions (e.g., NO_*x*_) that could lead to adverse health impacts in downwind populations.

### Limitations

We used power plant emissions data from 2020 to 2023 to scale year 2020 coal SO_2_ contributions to PM_2.5_ derived with the CMAQ-DDM and HyADS models. This introduces uncertainty since emissions from both coal power plant and other sources have changed across these years; notably, NO_*x*_ emissions have decreased, which likely changed PM_2.5_ response to EGU SO_2_ emissions in unique ways across the country.^34^ EPA designates the design value of a county based on the monitor within the county with the highest concentration. Our analysis uses area-weighted county-wide EGU PM_2.5_ averages, which are reasonable given overall spatially homogeneous PM_2.5_ contributions from SO_2_ emissions.^32^ In a previous analysis, we found that despite the COVID-19 pandemic impacting mobile emissions in 2020, SO_2_ emissions from EGUs saw an increase compared to the business-as-usual scenario,^54^ suggesting that using year 2020 as a baseline is reasonable for studying coal air quality impacts. Additional uncertainty is introduced in the estimates of mortality avoided because baseline mortality rates for the Medicare population are assumed constant; however, a previous analysis found little variability in that rate in recent years.^7^

### Implications

Emissions controls implemented under the Clean Air Act and technological advances have led to PM_2.5_ concentrations reductions across the eastern United States. Our results show that further reductions in EGU SO_2_ emissions could help bring nonattainment areas in the eastern and central U.S. into compliance with the new PM_2.5_ NAAQS. The list of counties that could be brought into attainment by reducing EGU SO_2_ emissions does vary between years (Figure S7), and it is sensitive to the accuracy of CMAQ-DDM. Still, if the PM_2.5_ reductions available through EGU SO_2_ emissions reductions is substantially underestimated by CMAQ, 6 out of the 9 counties identified in the primary analysis would have been able meet the 9.0 μg/m^3^ standard through EGU SO_2_ reductions alone.

Other routes beyond coal SO_2_ emission reductions are available for reducing ambient PM_2.5_ concentrations. EPA’s analysis supporting the 2024 PM_2.5_ NAAQS focused primarily on primary PM_2.5_ emissions reductions (e.g., from industry and transportation sources) in each county and in adjacent counties.^14^ PM_2.5_ from wildland fires has slowed or reversed decades-long progress toward achieving NAAQS across much of the United States,^55^ but mitigating ambient pollution from fire sources is challenging due to long-distance transport and uncertain relative benefits of strategies like prescribed burning.^56^ Secondary inorganic PM_2.5_, consisting of sulfate, nitrate, and ammonium, was reduced substantially through SO_2_ and NO_*x*_ emissions reductions since the 1990s.^57^ Because it is unlikely that ammonia concentrations are limiting secondary inorganic PM_2.5_ formation in most areas of the country,^58,59^ continued SO_2_ and NO_*x*_ emissions reductions will likely be more effective at reducing secondary inorganic PM_2.5_ than targeting NH_3_ emissions.

Beyond achieving attainment, reduced emissions from coal power plants offers substantial public health and climate benefits that extend beyond nonattainment counties. Renewable alternatives to coal in power generation have become increasingly competitive, often making new renewable capacity more cost-effective than operating existing coal plants.^60^ This suggests the potential to replace the generating capacity with alternatives that do not emit greenhouse gases or air pollutants. We estimated over twice as much savings in lifetime CO_2_ emissions achievable by replacing the identified coal plants with wind or solar compared to replacement with natural gas.

The timeline and enforcement of currently enacted air quality and climate mitigation regulations remain uncertain under the current administration, with ongoing legal challenges and potential implementation delays. Nevertheless, near-term strategies to reduce emissions and shorten coal plant life spans remain an avenue for meeting established standards and achieving public health and climate benefits. The rapid growth in electricity demand—driven by expanding data centers and electric vehicle charging infrastructure—poses both challenges and opportunities. While there is evidence that increased electricity demand and political pressure has led utilities to consider delaying the retirement of coal power plants,^61^ our analysis strengthens the case for accelerated investments in renewable energy, grid modernization, and energy efficiency.

## Supplementary Material





## Data Availability

The data supporting the analysis is provided as .

## Notes

Additional details on CMAQ evaluation and other results (PDF)
Co-benefits associated with shuttering each coal facility, with benefits split into those expected under facilities’ current retirement plans vs those facilities with no retirement plans (CSV)
