# Supplementary material for: Emissions Reductions at Coal Power Plants Continue to Offer Routes to Meet New US PM2.5 Standards
Source: Environ Sci Technol. 2026 May 5;60(19):13796–803. doi: 10.1021/acs.est.5c13420 (PMC13192323; doi:10.1021/acs.est.5c13420)
Supplement: Supplementary file 1 [file es5c13420_si_001.pdf]

Emissions Reductions at Coal Power Plants Continue to Offer Route to Meet New US $PM_{2.5}$ Standards

Munshi Md Rasel[1], Daniel S. Cohan[2], Daniel Tong[3], and Lucas R.F. Henneman[1*]

[1]Department of Civil, Environmental, and Infrastructure Engineering, George Mason University, Fairfax, VA 22030, United States

[2]Department of Civil and Environmental Engineering, Rice University, Houston, TX 77005, United States

[3]Atmospheric, Oceanic and Earth Sciences Department, George Mason University, Fairfax, VA 22030, United States

Supplemental Information
9 pages
6 figures
1 table

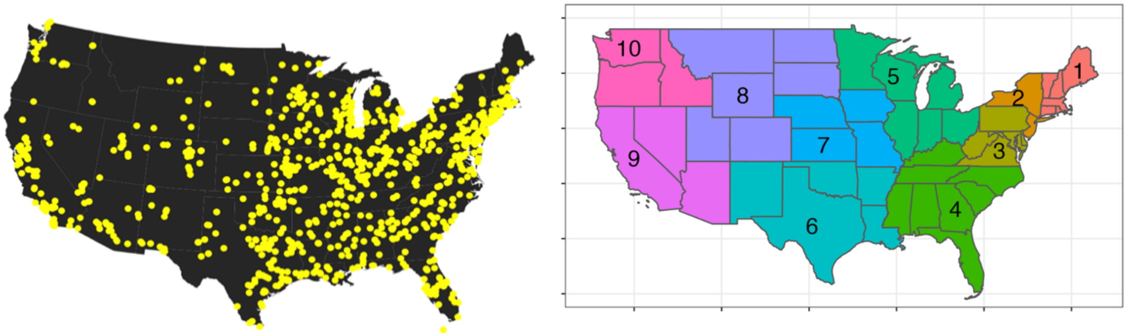

Figure S1: Contiguous United States domain for the CMAQ model run at 12 km × 12 km resolution, overlaid with coal EGU locations in 2020 (left). At right, the 10 region definitions used for CMAQ model evaluation.

CMAQ Model Evaluation

The CMAQ model's performance against daily ground observations for $PM_{2.5}$ (545 AQS sites) was evaluated using criteria from [35]. Nationwide, the model shows a normalized mean bias (NMB) of -23% and a normalized mean gross error (NMGE) of 32%, both within acceptable ranges. Regionally, the model generally meets the NMB criteria of +/-35% and NMGE criteria of <50%, with notable underpredictions in Regions 8 and 10. These findings align with [21] study, suggesting robustness in the underlying emissions data. However, 2020 posed unique challenges due to COVID-19 and wildfire emissions, introducing uncertainties in emissions projections. Despite these challenges, the CMAQ model shows strong overall performance in predicting $PM_{2.5}$ concentrations.

Evaluation metrics

CMAQ model output is evaluated with respect to EPA's ground observations data using evaluation metrics such as Normalized Mean Bias (NMB), Normalized Mean Gross Error (NMGE), Mean Bias (MB) as used in other studies [35,45]:

$$\boldsymbol{NMB} = \frac{\sum(\boldsymbol{P_i}-\boldsymbol{O_i})}{\sum \boldsymbol{O}_{\mathbf{i}}} \qquad \textit{Equation S-1}$$

$$\boldsymbol{NMGE} = \frac{\sum|\boldsymbol{P_i}-\boldsymbol{O_i}|}{\sum \boldsymbol{O_i}} \qquad \textit{Equation S-2}$$

$$\boldsymbol{RMSE} = \sqrt{\frac{\sum(\boldsymbol{P_i}-\boldsymbol{O_i})^2}{\boldsymbol{n}}} \qquad \textit{Equation S-3}$$

Here, O_i is i^{th} observation, P_i is i^{th} model simulated value, n is number of observations-simulation pairs. NMB and NME provides deviation from actual as percent having a lower and upper bound of $\pm$100% for NMB and -100% to 0% for NME. NMB and NME will help to understand how big the error is of the mean observation.

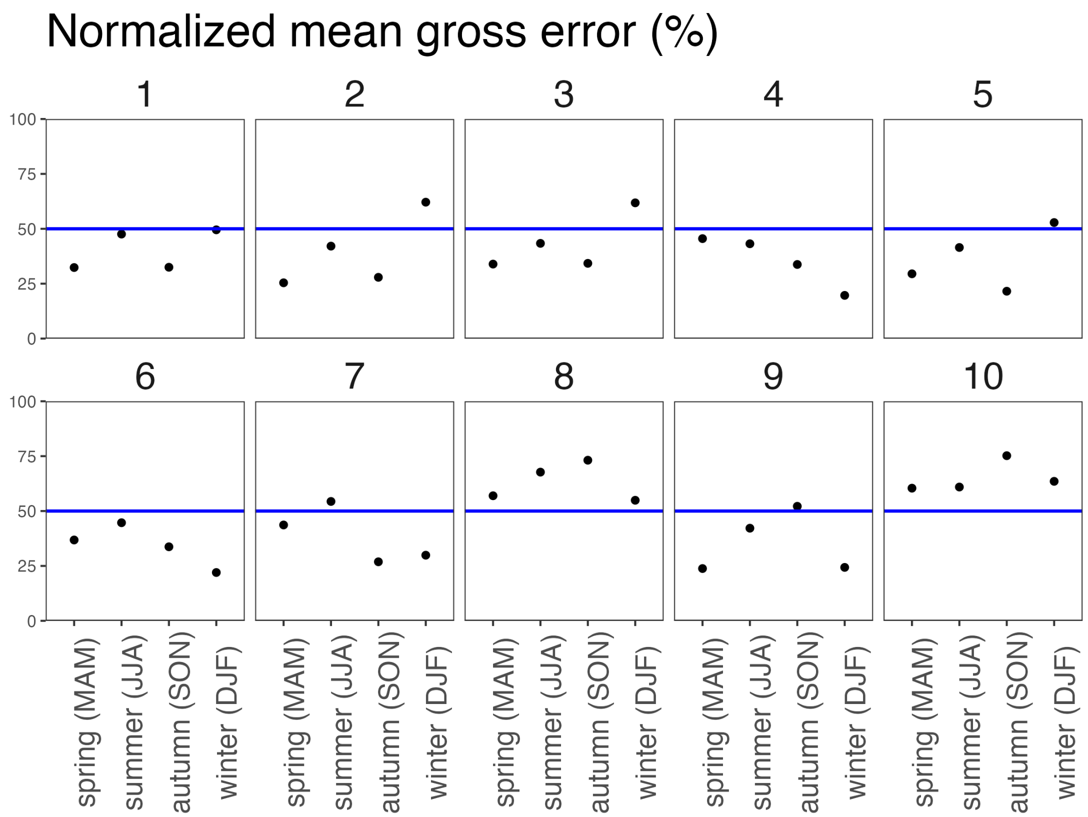

Figure S2: Normalized mean gross error for each EPA regions and for different seasons. Regional map can be seen in Figure S1. Criteria line is based on Emery et al. (2017)[35].

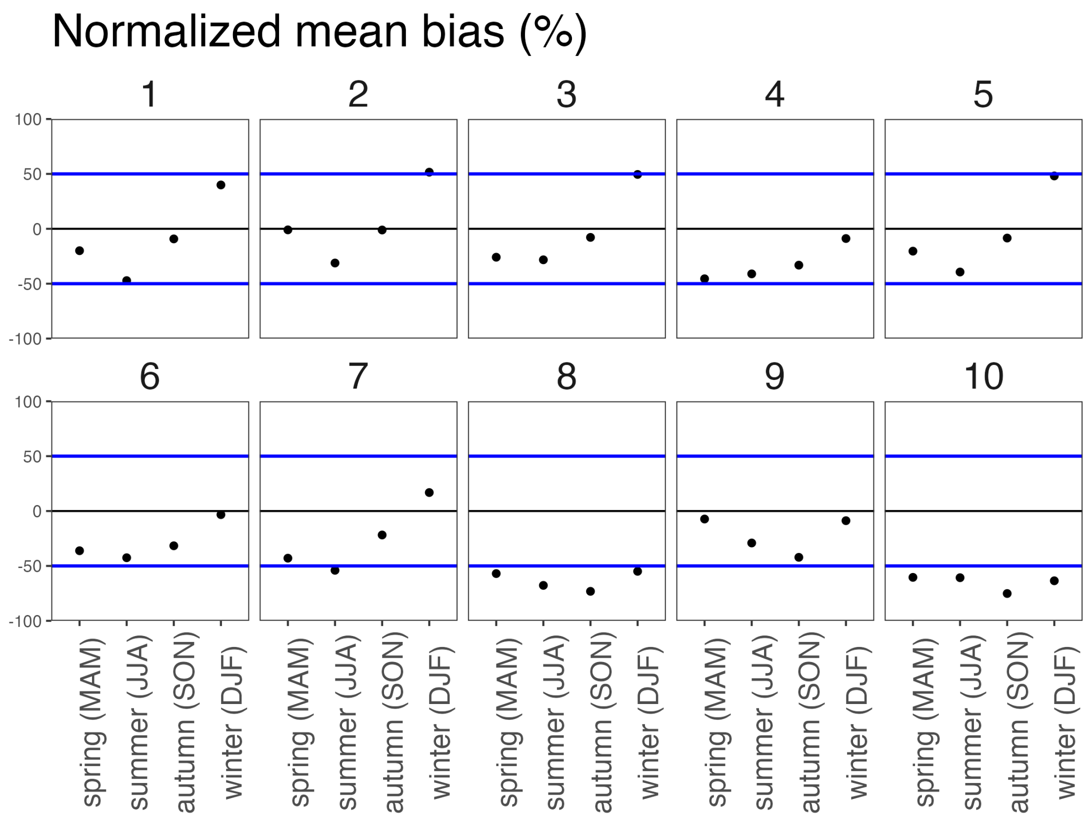

Figure S3: Normalized mean bias for each EPA regions and for different seasons. Regional map can be seen in Figure S1. Criteria line is based on Emery et al. (2017)[35]

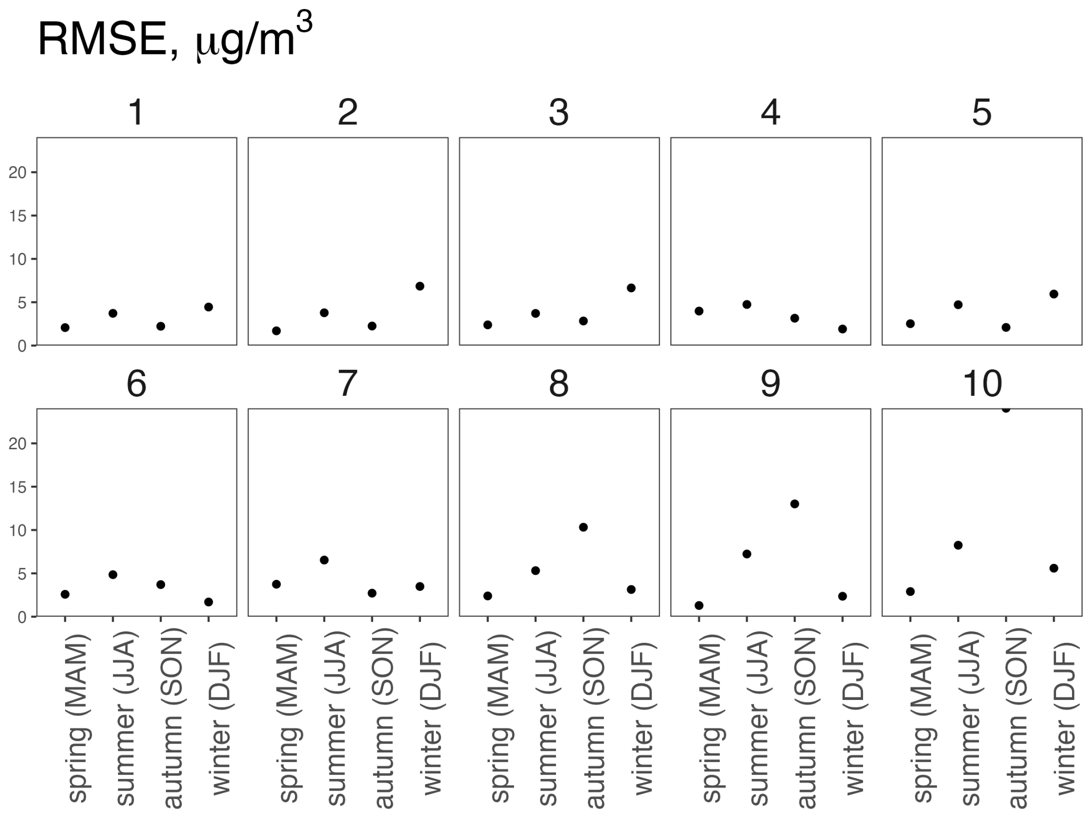

Figure S4: Root mean square error (RMSE) for each EPA regions and for different seasons. Regional map can be seen in Figure S1.

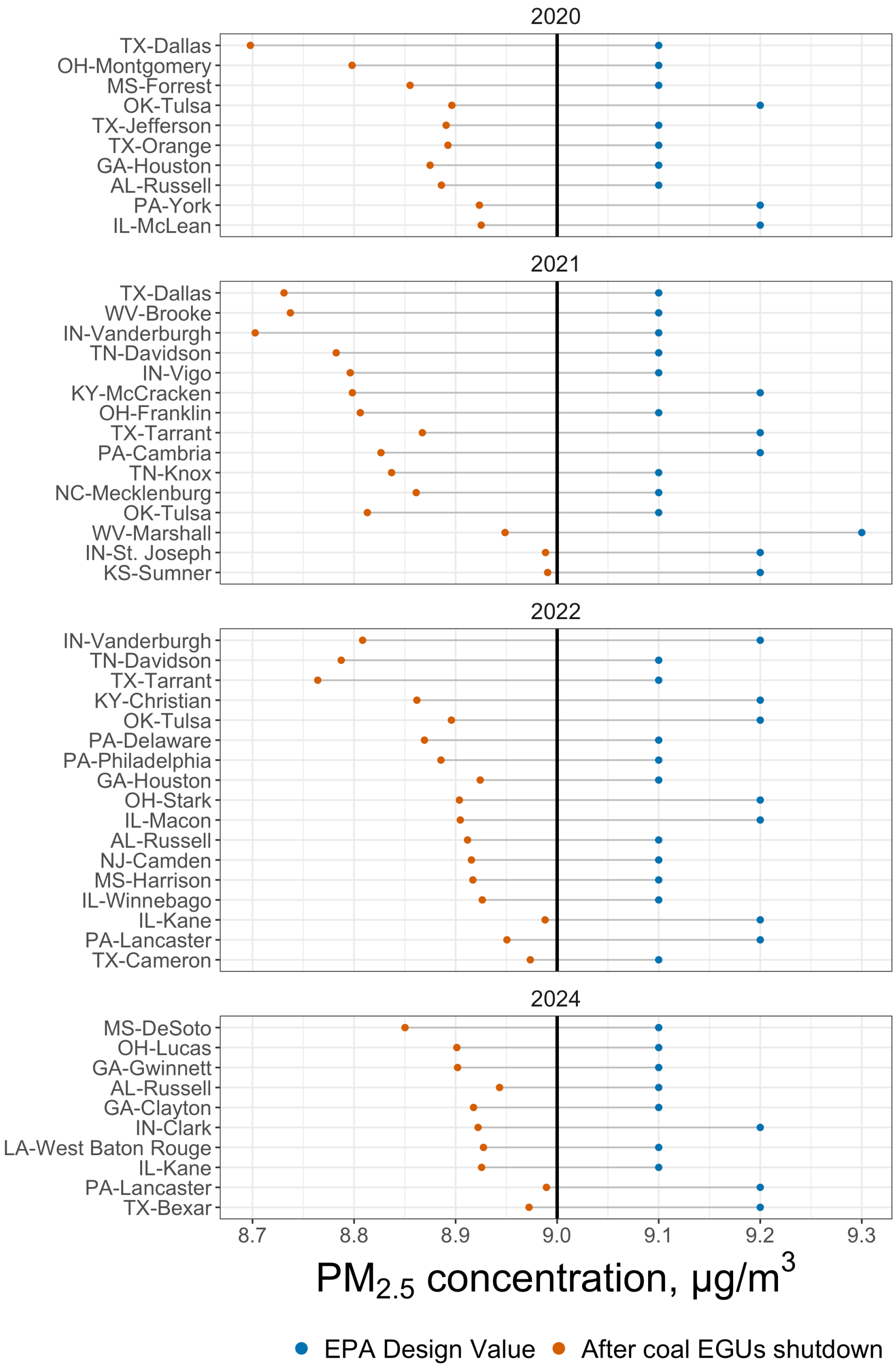

Figure S5: Observed and expected design values with a 100% reduction in EGU SO_2 emissions as calculated from the year 2020 CMAQ-DDM sensitivities adjusted to years that contribute to each design value by the ratio of facility-specific HyADS. Counties are restricted to those that could attain the 9.0 μg/m^3 standard through EGU SO_2 emissions reductions alone.

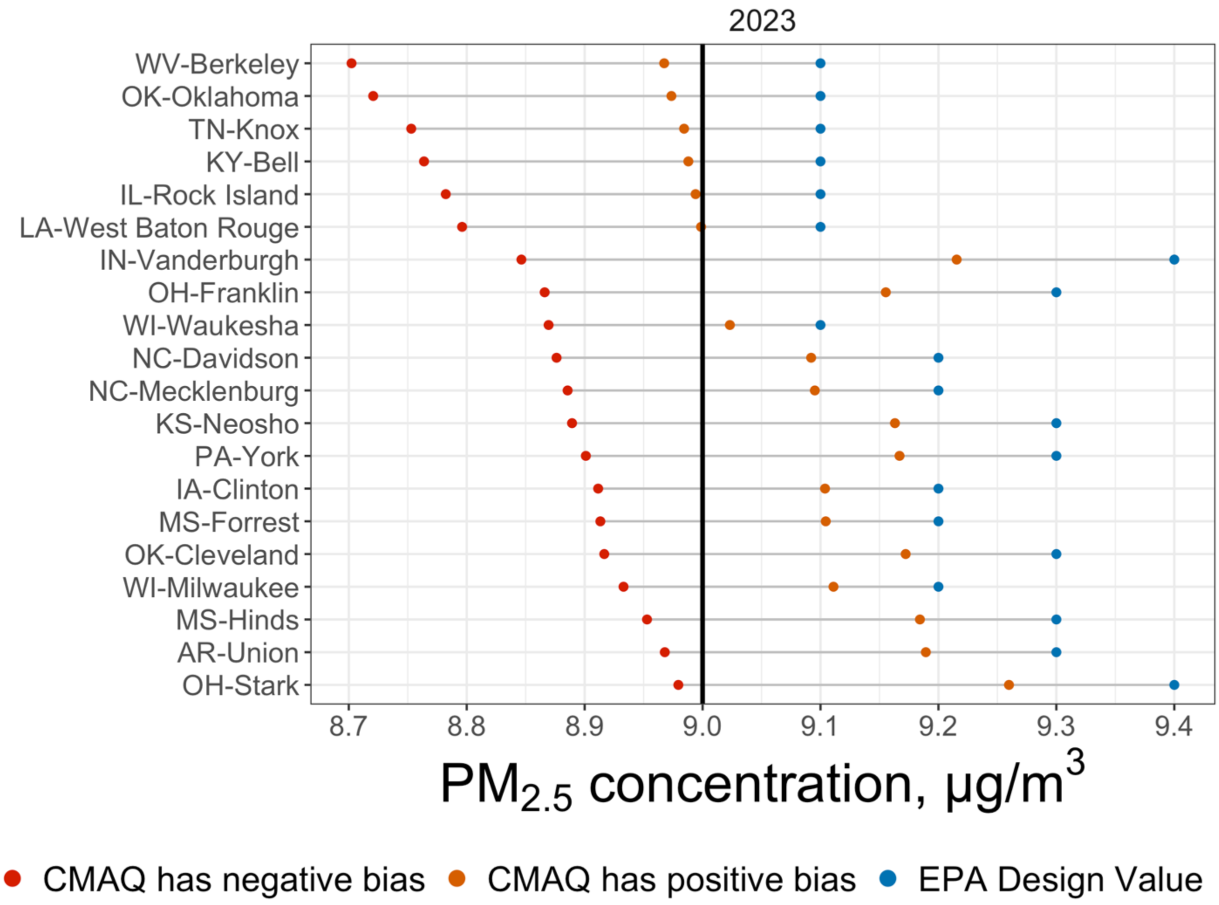

Figure S6: Sensitivity analysis: year 2022 observed and expected design values with a 100% reduction in EGU SO_2 emissions as calculated from the year 2020 CMAQ-DDM sensitivities adjusted to years that contribute to each design value by the ratio of facility-specific SO_2 emissions. Counties are restricted to those that could attain the 9.0 μg/m^3 standard through EGU SO_2 emissions reductions alone. "CMAQ has positive bias" corresponds to the case when CMAQ-DDM $PM_{2.5}$ sensitivities are multiplied by 50%; "CMAQ has negative bias" corresponds to the case when CMAQ-DDM $PM_{2.5}$ sensitivities are multiplied by 150%.

Table S1: For US balancing authorities, total capacity (GW) for coal facilities identified as needing to shutter to meet the NAAQS at selected counties, total capacity in facilities in each balancing authority, and the fraction of total capacity from the identified units. Data is from the 2023 eGRID dataset, and only considers units with capacity factor greater than 0.

Balancing authority	Capacity of units impacting non-attainment areas (GW)	Total electric utility coal capacity (GW)	Fraction of total capacity from identified units
AECI	2.5	6.7	0.37
CPLE	2.6	18	0.14
DUK	2.1	27	0.08
ERCO	12	140	0.08
FMPP	1.3	3.7	0.34
LGEE	6.5	9.9	0.66
MISO	26	196	0.13
NWMT	1.6	3.6	0.46
PACE	3.1	16	0.20
PJM	28	220	0.13
SC	2.4	5.4	0.44
SCEG	1.4	8.0	0.17
SEC	2.6	3.5	0.74
SOCO	5.5	70	0.08
SPA	0.3	2.5	0.10
SWPP	8.8	99	0.09
TEPC	1.8	3.6	0.49
TVA	5.6	43	0.13
WACM	1.2	9.7	0.13
Total	115	883	0.13
